# From margins to mainstream: reimagining oral health in integrated healthcare

**DOI:** 10.3389/fdmed.2026.1905156

**Published:** 2026-08-21

**Authors:** Melanie Aley, Salima Alibhai, Amanda Bloomfield-Gallie, Jamie Dooley, Gitana Rederiene, Adam Rogers, Roisin McGrath

**Affiliations:** 1 Sydney School of Dentistry, The University of Sydney, Sydney, NSW, Australia; 2 Faculty of Dentistry, The University of British Columbia, Vancouver, BC, Canada; 3 University of Lincoln, Lincoln, United Kingdom; 4 National Network of Healthcare Hygienists, Grand Rapids, MI, United States; 5 Vilnius University, Vilnius, Lithuania; 6 Institute of Clinical Dentistry, University of Oslo, Oslo, Norway; 7 Melbourne Dental School, The University of Melbourne, Parkville, VIC, Australia

**Keywords:** integrated healthcare, interprofessional collaboration, oral health, oral health professionals, prevention

## Abstract

Oral health remains persistently marginalised within mainstream healthcare despite the global burden of preventable oral diseases and growing recognition of the need for integrated, person-centred care. This perspective paper examines the structural, professional, and educational barriers that continue to separate oral health from broader health systems, including limited interprofessional collaboration, fragmented service delivery, and underutilisation of the oral health practitioner workforce. Drawing on international policy directions and emerging models of integrated care, the paper argues that oral health practitioners are well positioned to support prevention, early intervention, health promotion, and care coordination across healthcare settings. The authors highlight the importance of workforce utilisation, interprofessional education, policy alignment, and system-level collaboration in advancing equitable access to oral healthcare. Through a globally informed perspective, this paper proposes practical priorities for embedding oral health within integrated healthcare systems and repositioning oral health as a fundamental component of overall health and wellbeing.

## Introduction

Oral health has been persistently sidelined in health policy, not for lack of research evidence, but due to historical and structural separation from mainstream healthcare. Oral diseases affect 3.5 billion people worldwide and are among the most prevalent non-communicable diseases (1). This marginalisation is increasingly at odds with global commitments to integrated health systems and universal health coverage (1, 2). Preventable oral conditions - including caries, periodontal disease, and oral cancers - have substantial impacts on quality of life, nutrition, employability, and social participation, contributing to productivity loss and avoidable healthcare costs (2).

The marginalisation of oral health stems from the long-standing organisation of dentistry as a treatment-focused, often private service that evolved separately from medical and public health systems. This separation has entrenched professional and organisational silos, limiting prevention, early intervention, and interprofessional collaboration. Consequently, oral healthcare access remains uneven and inequitable; cost, transportation, and service design disproportionately affect older adults, low-income individuals, people living in rural/remote areas, Indigenous communities, and individuals with chronic health conditions (1, 3).

Over the past decade, global policy has increasingly recognised that marginalisation must be addressed, and that isolated dental systems are inadequate. The WHO has explicitly called for the integration of oral health into primary healthcare and Universal Health Care (UHC), and policy frameworks increasingly advocate for prevention, early detection, interprofessional collaboration, and workforce reform (3, 4).

Within this emerging integration agenda, dental hygienists, dental therapists and oral health therapists (collectively referred to here as oral health practitioners [OHPs], noting variation in nomenclature and scope across jurisdictions) are pivotal to reducing marginalisation and translating policy into equitable, effective models of care. Their prevention-focused scope, expertise in health promotion, and ongoing patient management position them to integrate core activities such as screening, risk assessment, behavioural counselling, referrals, and care coordination across healthcare settings, all of which are consistently identified as essential to effective medical–dental integration (5, 6).

This paper argues that the oral health system marginalisation is solvable and that the existing OHP workforce is central to the solution. Throughout this paper, we distinguish between empirically demonstrated outcomes, policy-driven recommendations, and forward-looking projections based on systems-level reasoning and emerging implementation evidence. We identify common barriers and enablers and outline workforce, education, and policy priorities aligned with the WHO's renewed oral health agenda (1, 6).

## Why oral health remains siloed

Integrating oral health into mainstream healthcare requires understanding existing barriers. One core obstacle is the lack of interprofessional collaboration (IPC) with OHPs. IPC is widely recognised as a foundational component of integrated, patient-centred healthcare, with strong evidence for improving collaborative processes, although its direct impact on clinical outcomes is less consistently demonstrated (7). The WHO defines IPC as multiple health professionals working collaboratively with patients, families, and communities to deliver high-quality care (8). In oral health, effective IPC should extend beyond the dental team to involve nurses, general medical practitioners, allied health professionals, and community-based professionals. Despite strong conceptual support, IPC remains limited in practice, exposing a persistent gap between policy rhetoric and operational reality.

While interprofessional education and collaborative practice are widely promoted as enablers of integrated care, it is important to distinguish between different types of outcomes reported in the literature. Much of the current evidence demonstrates improvements in provider knowledge, attitudes, and collaborative behaviours, as well as process-level outcomes such as referral pathways and communication (7). However, consistent evidence linking these changes to measurable patient or population-level health outcomes remains limited and context-dependent (9). As such, while interprofessional approaches are strongly supported conceptually and in policy frameworks, their direct clinical impact should be interpreted with appropriate caution.

Existing barriers are systemic and span professional, organisational, and educational domains. Professionally, limited oral health literacy among non-dental professionals and poor understanding of OHPs’ scope and competencies create unclear role boundaries (10). Oral health is frequently perceived as technically complex, procedure-oriented, or outside of general healthcare, discouraging engagement in screening, prevention, and referral. Lack of shared terminology or shared care protocols further hinder communication and continuity of care (11).

Organisational and educational barriers further reinforce this fragmentation. Dental services are typically delivered separately from hospitals and primary care, with limited co-location, incompatible data systems, and funding models that prioritise episodic treatment over team-based prevention (6, 12). Educational silos further compound these problems. Oral health is often scant or absent in medical, nursing, and allied health curricula, resulting in low confidence to recognise oral diseases or manage referral processes (13). Dental education programmes likewise focus on discipline-specific technical competence, with limited exposure to interprofessional practice in diverse settings. Terminology matters; “dental health” reinforces a narrow, treatment-oriented paradigm, whereas “oral health” is broader, aligning with prevention, wellbeing, and chronic disease management (14). These structural and educational constraints interact to sustain siloed practice, meaning that incremental or isolated reforms are unlikely to achieve meaningful integration. Importantly, these barriers are interdependent: professional role ambiguity, organisational separation, and educational silos reinforce one another, creating a cycle that limits both collaboration and system-level change.

Additional barriers include low oral health literacy and limited recognition of oral-systemic health links. Periodontal disease is increasingly associated with diabetes, cardiovascular disease, and adverse pregnancy outcomes, yet early detection outside dental settings remains inconsistent (15). Dental caries, though largely preventable, is seldom identified during general healthcare encounters (16). Awareness of and screening for oral cancer is also inadequate, constrained by limited training and unclear professional responsibility (17).

## Untapped potential of the oral health workforce

OHPs represent a critical, and currently underleveraged, enabler of integrated, prevention-oriented oral healthcare (18, 19). Emerging evidence suggests these practitioners can safely and effectively deliver oral health education and care across broader healthcare settings (13, 20).

Embedding OHPs within general healthcare services can strengthen referral pathways, with audits and pilot studies indicating improvements in coordination and earlier identification of oral disease (21, 22). This integration is particularly important for populations with complex needs, including older adults, people with chronic disease, and those receiving residential or home-based care. Yet regulatory restrictions, funding models, and professional hierarchies continue to constrain full-scope practice and reinforce dentist-centric models that are poorly aligned with prevention and equity goals (23, 24).

Realising workforce potential requires moving beyond mere task substitution to genuine integration, with OHPs embedded as accountable members of multidisciplinary teams. This requires scope-of-practice reform, funding models that reward team-based preventive care, and interprofessional education across undergraduate, postgraduate, and continuing professional development pathways (25). Without coordinated, system-level change, integration will remain fragmented and unsustainable. This underutilisation reflects system failure, not workforce capability. In many settings, policy and funding structures remain misaligned with integration goals, continuing to prioritise treatment-focused models over collaborative, prevention-oriented care.

## Recognising and strengthening an integration-ready workforce

OHPs are highly educated and regulated, practising within nationally defined scopes, competency frameworks, and professional standards. Across jurisdictions they demonstrate expertise in prevention and clinical care. The challenge is therefore not one of foundational training or professional legitimacy, but ensuring their competencies are recognised, appropriately extended, and effectively mobilised within integrated healthcare systems. However, workforce capability does not automatically translate into system readiness. Integration is shaped by financing structures, regulatory frameworks, professional boundaries, and organisational capacity. Without alignment across these domains, even well-prepared workforces may remain underutilised within fragmented systems.

Looking ahead, OHP roles can be bolstered through globally recognised post-licensure credentials. Credentials in areas such as hospital oral care, gerontology, oncology, and special care dentistry would both enable career progression and give health systems transparent signs of capability and accountability in integrated care settings (7, 26).

Within this context, there is an opportunity to formalise advanced interprofessional capabilities. Micro-credentials offer verifiable, stackable recognition of competencies and professional development. When aligned with international standards for program certification and accreditation, these approaches provide quality assurance and portability (27, 28). For oral health, such systems could support accelerated workforce upskilling in response to evolving population needs, service models, and digital health innovations.

Professional self-governance is central to this vision, not as a protectionist claim, but as a prerequisite for agile, responsive workforce development in rapidly evolving health systems. Jurisdictions where OHPs are active contributors to their own regulatory and credentialing frameworks are best positioned to respond to system change and drive innovation. This approach aligns directly with the WHO Global Oral Health Action Plan, which emphasises strengthened regulation, education, and workforce development as foundational to oral health reform (4).

## Global snapshots of integration efforts

Despite their capability, systemic barriers shape OHP utilisation. Nonetheless, international examples illustrate how integration can be achieved in different health systems. Variation lies in how systems regulate, fund and integrate the workforce. To support comparison, each jurisdiction is considered across four key dimensions: 1. governance and regulation, 2. financing structures, 3. scope of practice, and 4. mechanisms for integration. This framework highlights how system design shapes the degree to which oral health is embedded within broader healthcare.

### Australia

Dental hygienists, dental therapists and oral health therapists are registered with the Dental Board of Australia and practise as autonomous, independent clinicians within defined education, training and competence frameworks, and according to their division of registration (29). National regulation under the Health Practitioner Regulation National Law provides consistent registration, continuing professional development and baseline competence requirements. However, state and workplace variations in clinical privileges, prescribing and delegation create jurisdictional differences that limit uniform deployment.

The Australian oral health therapist combines qualifications in dental therapy and dental hygiene and focuses on oral health across the lifespan. Their clinical scope includes assessment, diagnosis, treatment, management and prevention — encompassing preventive care, restorative procedures, simple extractions and periodontal treatment — and they work across private and public clinics, community health centres, schools, aged care and Aboriginal Community Controlled Health Organisations. Workforce maldistribution, limited referral pathways and other system barriers mean this capacity is often underused, constraining access and increasing pressure on specialised services.

Oral healthcare financing is fragmented: private fee for service, limited state/territory public dental services for priority groups, and targeted federal programs (30). These arrangements reinforce episodic, treatment centred care and leave few sustainable payment pathways for OHPs working outside traditional clinics. From July 2022, OHPs have been able to access Medicare provider numbers to claim under the Child Dental Benefits Schedule; this change was also the catalyst enabling OHP claims through a growing number of private health insurers (31).

The University of Melbourne ‘Eyes, Ears and Mouth’ program demonstrates how multidisciplinary screening - with oral health students working collaboratively with audiology, optometry, and speech pathology students - can embed essential screening and prevention services within community settings. Such place-based models demonstrate that OHPs can act as integrative practitioners within holistic, prevention-oriented care models, challenging the notion that dentistry must remain clinic-bound. They also create valuable opportunities for interprofessional education and early intervention. However, these examples are often implemented at pilot or local levels, and their scalability remains uncertain without sustained funding, policy alignment, and system-level infrastructure to support wider adoption.

The Australian context reflects a system where regulatory recognition exists, but financing and structural fragmentation limit integration at scale.

### Canada

In Canada, dental hygienists are autonomous, regulated health professionals whose scope encompasses prevention, health promotion, education, administration, research, and public health practice (32, 33). Dental hygiene has been formally recognised as one of twelve public health professions within the Pan-Canadian Framework for Public Health Human Resources Planning (34), acknowledging its contribution beyond chairside care. This recognition has supported expanded roles for dental hygienists in community health, long-term care, health promotion programs, and Indigenous health services, although access to these roles varies by province.

National frameworks for interprofessional education, most recently updated in 2024, alongside accreditation standards requiring interprofessional learning experiences, have further embedded collaborative practice within Canadian dental hygiene education (35, 36). These educational foundations create favourable conditions for integration with primary healthcare. Dental care remains largely excluded from publicly funded health insurance for adults, despite the recently implemented Canadian Dental Care Plan (37, 38), limiting the extent to which integration can translate into universal access. As a result, despite a well-prepared workforce, structural financing constraints continue to restrict system-wide impact. This contrast illustrates that workforce readiness alone is insufficient; without aligned financing and coverage, integration cannot achieve population-level impact.

This illustrates strong workforce and educational integration capacity constrained primarily by financing structures.

### Europe

Across Europe, oral health systems vary considerably in governance and regulation, financing models, scope of practice, and the degree of integration with broader healthcare systems, contributing to persistent inequalities in access to care (39). Regulatory frameworks differ in how they define professional autonomy, education, and workforce responsibilities. In countries with supportive legislation, dental hygienists contribute independently to preventive care within multidisciplinary teams, whereas historical models continue to restrict collaboration and workforce flexibility (40). Financing models range from publicly funded preventive programmes to mixed or predominantly private systems. Greater public investment in prevention facilitates integration with primary care, school health, maternal and child health, and long-term care, while private financing may limit equitable access. The scope of practice of dental hygienists also varies widely. In several countries, they provide education, prevention, periodontal maintenance, risk assessment, and screening, while restrictive legislation limits these roles elsewhere.

The Platform for Better Oral Health in Europe has documented multiple programmes integrating oral health into broader prevention strategies, with evaluations and pilot initiatives indicating potential for improved access and prevention across the life course. These include school-based programmes, maternal–child health initiatives, and chronic disease prevention models, which exemplify oral health integration in practice through the involvement of dental hygienists delivering education, prevention, and screening within multidisciplinary teams (41). While these initiatives illustrate integration in practice, many remain context-specific, and evidence supporting large-scale implementation across health systems is still evolving.

This demonstrates how variability in governance and scope of practice directly influences integration capacity.

### United Kingdom

In the United Kingdom, the scope of practice for OHP's is developed and regulated by the General Dental Council across the four nations (42). There are two oral health systems: a publicly funded system, the National Health Service (NHS), where patients pay a contribution to care or are exempt from charges, and a private system utilising insurance and out-of-pocket payments. In both systems, access and integration challenges persist and levels of successful integration are unknown.

Workforce shortages and long-standing constraints within the NHS Dental contract have significantly reduced contract holders, directly impacting patient access to routine care. In response, policy reforms have sought to optimise interprofessional working by expanding the roles of dental hygienists and therapists, enabling them to work to their full licence and initiate and complete courses of care to increase output and integration, and since 2024, have permitted the direct supply and administration of some prescription-only medicines through an exemption to prescribing framework (43).

Historically, contractual arrangements have prioritised dentist-led activity and treatment targets over prevention and team collaboration, and this is recognised. The NHS dental contract has been subject to recent reform and from July 2026, complex care pathways will be funded to enable time for diagnosis, behaviour change, prevention treatment and stabilisation of caries and periodontal conditions and incentives for fissure sealing and fluoride application (44, 45). The 10-year health plan for England, released in October 2025, aims to co-locate dental therapists into community health hubs to widen access and target inequalities (46). While regulatory change has created opportunities for integration and operationalisation, there are organisational, educational and cultural barriers that remain and inhibit full realisation of interprofessional delivery of oral care, particularly for underserved populations and in rural and coastal geographies in the United Kingdom.

However, targeted policies and implementation frameworks, such as Scotland's Childsmile initiative and the Mouth Care Matters program, illustrate how integration can be operationalised through strong public health leadership, cross-sectoral collaboration, and workforce development (47, 48). These initiatives demonstrate that when oral health is positioned as a shared responsibility within health systems, substantial improvements in access, prevention, and equity are achievable.

This highlights partial regulatory progress without fully aligned financing and organisational systems.

### United States of America

The registered dental hygienist (RDH) is the primary preventive oral health provider in the United States, with more than 220,000 currently licensed under state dental practice acts that vary in supervision requirements and scope of practice authority (49). Dental therapy is authorized in 14 states, with approximately 200 dental therapists actively practicing nationwide (50). According to the American Dental Hygienists’ Association (ADHA), the dental hygienist functions as a primary care oral health professional whose practice centers on the full cycle of patient-centered preventive care — from assessment and diagnosis through planning, implementation, evaluation, and documentation — and is designed to be delivered collaboratively within the broader healthcare team (51). While this scope is consistent nationally, governance and supervision requirements are determined at the state level, resulting in significant variation across jurisdictions. Since 2000, the proportion of states authorizing direct access — permitting RDHs to initiate care without the physical presence or prior authorization of a dentist — has increased from 18% to 84%, and in 2026 the ADHA formally adopted a Full Practice Authority policy supporting autonomous practice across all U.S. jurisdictions (49, 52, 53).

Financing of dental hygiene services has historically been routed through supervising dentists within the fee-for-service private practice model, though a subset of states now authorize direct Medicaid reimbursement for dental hygiene services delivered in public health clinics, schools, and alternative care settings (52). Integration of oral health into broader healthcare delivery is most advanced in the public health sector, where Federally Qualified Health Centers and community health centers collectively deliver 16.8 million dental visits annually and represent the largest system of integrated primary and dental care in the nation, though disconnected EHR systems, absent referral pathways, and fee-for-service payment models have been documented as barriers to advancing integration beyond these settings (54, 55).

This reflects a highly variable system where integration depends heavily on state-level regulatory and financing conditions.

## Vision 2030: a framework for action

This section presents a forward-looking framework synthesising current global policy trajectories and system reform priorities. It is intended as an aspirational agenda rather than a prediction or a guarantee of specific outcomes. The coming decade is expected to require substantial reform of dental systems, driven by demographic shifts, widening inequities, and the constraints of fee-for-service models. Reform is necessary to meet current and future population needs (56). Priority actions commonly identified in the policy literature include greater integration of oral health services into broader health systems; a shift from treatment-dominated models to prevention-focused care; increased emphasis on equity and cost-effectiveness; and new approaches to detection, surveillance and evaluation of oral disease (Box 1). These changes are expected to reposition oral health as a core to patient care and safety in routine clinical workflows, multidisciplinary care pathways, and system performance frameworks. However, the feasibility of these reforms is contingent on addressing persistent structural barriers, including misaligned funding models, regulatory constraints, and limited system capacity for scaling local innovations.

Box 1 **Key actions for integrating oral health into mainstream healthcare.**

Achieving this transformation is likely to require coordinated action across five key domains:

- Embed oral health within routine care pathways
- Integrate OHPs into multidisciplinary teams
- Prevent disease through risk-based, life-course care
- Connect systems through shared data and digital infrastructure
- Enable equity through policy, funding, and accountability mechanisms

### Increased integration of oral healthcare

To strengthen the capacity and resilience of oral health systems, oral health needs to be more fully integrated into primary and general health services (4, 57). The benefits of dismantling siloed practice to improve care coordination are long established (2, 6, 58, 59). Empowering OHPs to serve as collaborative, accountable members of multidisciplinary teams across primary care, community and institutional settings is a key strategy for strengthening integrated care models (57).

Enhanced collaboration and broader population reach enable better data collection, earlier identification of oral health problems, and timely referrals (60). Embedding oral health monitoring within general health settings also enables evaluation of service effectiveness, supports continuous quality improvement, and has the potential to contribute to reductions in the overall burden of oral disease (57, 61).

Initiatives such as incorporating oral health screening into child health checks may also be extended to additional periods across the life course and involve a broader range of non-dental providers (58). Such assessments could become standardised components of routine care pathways, including admission screening, pre-operative assessment, and chronic disease management. Achieving this will require alignment of general and oral health agendas (59) and strengthened interprofessional education (62). While evidence is still emerging in this field, OHPs are well positioned take on these more holistic, integrated role within the broader healthcare system, recognising and responding to patients’ overall health needs. Dental services are likely to become more embedded in hospital and emergency settings, with dental emergencies managed as acute conditions equivalent to other emergency presentations (63), and oral health recognised as integral to patient safety, particularly regarding infection and clinical complication risks.

Incompatible data systems remain a major barrier to integration, as dental services often rely on privatised platforms that do not interface with hospital records (6, 60). Integrating oral health into electronic health records has the potential allow oral health information to be routinely recorded, accessed and acted upon, shared across multidisciplinary teams, and used to support coordinated decision-making, continuity of care and population-level monitoring (12). Implementation is further complicated by entrenched professional hierarchies, resistance to role reconfiguration, and the slow evolution of financing and governance systems.

### Focus on prevention

As integration increases opportunities for engagement with oral healthcare, a parallel shift towards prevention should be prioritised (63). The predominance of curative, fee-for-service models has been widely criticised, particularly because the most prevalent oral diseases are largely preventable (2, 6). Integration has the potential to enable earlier and more consistent delivery of prevention across the life course, by embedding it in routine care pathways. Preventive advice and interventions will no longer be confined to dental clinics but be delivered in primary care, community services, and hospital settings.

Central to this shift is the adoption of standardised risk metrics and evaluation tools that extend beyond clinical disease indicators to capture behaviours, health literacy, attitudes, and environmental stressors. Such measures support a risk-based, life-course model of care embedded in routine clinical systems rather than isolated to dental settings (64, 65). Ongoing monitoring of risk trajectories is expected to assist both targeted prevention and service planning (6, 64). Historically, limited integration with behavioural science has constrained progress, although increasing collaboration with psychology is beginning to strengthen the evidence base, refine preventive interventions, and enhance policy relevance.

### Cost-effectiveness, equity, and sustainability

Greater integration and a prevention focus will likely drive increased scrutiny of cost-effectiveness, health equity, and sustainable care delivery (2, 63). Policy is expected to shift from promoting service availability toward ensuring utilisation and more equitable outcomes (6, 66). To support this shift, restructuring of the oral health workforce has been advocated, so practitioners increasingly operate at the highest level of their competence (56). Expanding the roles of OHPs has been proposed as a way to meet primary-care needs through ongoing risk assessment, preventive interventions, and active contribution to multidisciplinary management (2, 58).

Improving access to oral healthcare is especially urgent for older adults, where financial barriers, rising dependency and workforce shortages exacerbate inequities (62, 67). Over the next decade, scope-of-practice boundaries should be adjusted so practitioner skills match population needs, underpinned by public education to address misconceptions about emerging OHP roles (6, 56). Achieving this will depend on supportive policy and funding models that prioritise prevention and integrated care.

### Quality assurance

As scopes of practice evolve, demand will grow for transparent demonstration of competencies and robust quality assurance (2, 56). System-level evaluation of diagnostic accuracy, treatment outcomes, and preventive effectiveness should be scaled-up, and oral health indicators embedded within broader health system performance frameworks. Especially with technological advances such as the use of tele-dentistry to improve access and screening capacity (57, 60), caution must be taken to ensure the quality of equitability of care remains high. These developments should prompt questions about how training, accountability, and governance must adapt to support an already well-qualified oral health workforce as it integrates further and further into mainstream models of care.

## Conclusion

The continued marginalisation of oral health reflects structural and professional silos, not a lack of relevance to general health. Global policy momentum, emerging models of care and workforce innovation create a timely opportunity to move oral health from the margins to the mainstream of healthcare. OHPs are central to this shift; this integration-ready workforce is well positioned to deliver equitable, prevention-focussed care if supported by coherent education, regulatory reform, and strong professional leadership. Without coordinated, system-level reform, integration is likely to remain partial and inequities will persist.

## Data Availability

The original contributions presented in the study are included in the article, further inquiries can be directed to the corresponding author.
